# Concomitant Action of Structural Elements and Receptor Phosphorylation Determines Arrestin-3 Interaction with the Free Fatty Acid Receptor FFA4*

**DOI:** 10.1074/jbc.M114.568816

**Published:** 2014-05-09

**Authors:** Adrian J. Butcher, Brian D. Hudson, Bharat Shimpukade, Elisa Alvarez-Curto, Rudi Prihandoko, Trond Ulven, Graeme Milligan, Andrew B. Tobin

**Affiliations:** From the ‡ Medical Research Council Toxicology Unit, University of Leicester, Hodgkin building, Lancaster Road, Leicester LE1 9HN, United Kingdom,; § Molecular Pharmacology Group, Institute of Molecular, Cell and Systems Biology, College of Medical, Veterinary and Life Sciences, University of Glasgow, Glasgow G12 8QQ, Scotland, United Kingdom, and; ¶ Department of Physics, Chemistry and Pharmacy, University of Southern Denmark, Campusvej 55, DK-5230 Odense M, Denmark

**Keywords:** Arrestin, Diabetes, Fatty Acid, G Protein, G Protein-coupled Receptor (GPCR), Phosphorylation

## Abstract

In addition to being nutrients, free fatty acids act as signaling molecules by activating a family of G protein-coupled receptors. Among these is FFA4, previously called GPR120, which responds to medium and long chain fatty acids, including health-promoting ω-3 fatty acids, which have been implicated in the regulation of metabolic and inflammatory responses. Here we show, using mass spectrometry, mutagenesis, and phosphospecific antibodies, that agonist-regulated phosphorylation of the human FFA4 receptor occurred primarily at five residues (Thr^347^, Thr^349^, Ser^350^, Ser^357^, and Ser^360^) in the C-terminal tail. Mutation of these residues reduced both the efficacy and potency of ligand-mediated arrestin-3 recruitment as well as affecting recruitment kinetics. Combined mutagenesis of all five of these residues was insufficient to fully abrogate interaction with arrestin-3, but further mutagenesis of negatively charged residues revealed additional structural components for the interaction with arrestin-3 within the C-terminal tail of the receptor. These elements consist of the acidic residues Glu^341^, Asp^348^, and Asp^355^ located close to the phosphorylation sites. Receptor phosphorylation thus operates in concert with structural elements within the C-terminal tail of FFA4 to allow for the recruitment of arrestin-3. Importantly, these mechanisms of arrestin-3 recruitment operate independently from G_q/11_ coupling, thereby offering the possibility that ligands showing stimulus bias could be developed that exploit these differential coupling mechanisms. Furthermore, this provides a strategy for the design of biased receptors to probe physiologically relevant signaling.

## Teaser

**Background:** FFA4 is a receptor for long chain fatty acids and possible target for diabetes and inflammatory diseases.

**Results:** Sites of phosphorylation and interaction with arrestin-3 were mapped within the C-terminal tail.

**Conclusion:** Both phosphorylation and structural elements are required for interaction with arrestin-3.

**Significance:** Insight gained into arrestin-3 *versus* G protein signaling and implications for biased ligand development may drive identification of improved therapeutics.

## Introduction

There is growing public, scientific, and medical interest in the association between diet and well-being. This has been particularly so in the area of circulating free fatty acids, which are nutrients necessary to fuel cellular processes but more recently have been described as bioactive molecules that have direct impacts on biological processes by interacting with specific cell surface receptors (1). These free fatty acid receptors have now been identified as a family of G protein-coupled receptors (GPCRs),^4^ FFA1–4, that show selectivity based in part on the aliphatic chain length of fatty acids (2, 3). Hence, medium and long chain free fatty acids consisting of 6–12 carbons and more than 12 carbons, respectively, activate FFA1 (previously known as GPR40) and FFA4 (GPR120), whereas the short chain free fatty acids consisting of less than six carbons (such as acetate, propionate, and butyrate) activate FFA2 (GPR43) and FFA3 (GPR41) (2, 3). Interest in these receptors has centered on evidence that they play an important role in both metabolic processes, in particular glucose homeostasis and lipid metabolism, and inflammatory responses (4, 5). For this reason, members of this family of receptors are attractive drug targets for a number of human disease indications (6–8), in particular type 2 diabetes (9, 10).

Although to date the primary focus has been on the FFA1 receptor to which ligands have been developed and progressed to clinical trials for type 2 diabetes (9, 10), less is known of the action of the other fatty acid receptor that is activated by medium and long chain free fatty acids, namely FFA4. Although responding to the same natural fatty acids as FFA1, such as linoleic acid and “fish oils,” including ω-3 fatty acids, FFA4 shows limited sequence identity to the other members of the FFA family (2, 11). A primary role of FFA4 appears to be in the regulation of the release of the incretin glucagon-like peptide-1 from enteroendocrine cells in the gut (11). Through this mechanism, FFA4 may regulate insulin release and glucose homeostasis, although there is also evidence that FFA4 may be expressed on pancreatic cells and have a more direct role in insulin release (12). In addition, FFA4 appears to be expressed on adipocytes where this receptor is proposed to enhance glucose uptake and on macrophages where it mediates an anti-inflammatory response (13). Notably, the mechanisms of signal transduction utilized by FFA4 in these different tissues are distinct, in that G protein signaling via G_q_/G_11_ is the predominant pathway in enteroendocrine cells and adipose tissue, whereas the anti-inflammatory effects of FFA4 activation in macrophages proceed via the kinase TAK1 that is engaged through an arrestin-generated scaffold (11). It would therefore appear that targeting FFA4 would be of potential therapeutic value in the management of complications arising from obesity, particularly type 2 diabetes, and in the control of inflammation (5). That this may be important has recently been supported by studies that have provided a genetic link between a non-synonymous SNP in the human FFA4 coding sequence with the risk of obesity in man (14).

Until recently, synthetic ligands selectively targeting FFA4 have been lacking, and as such, many studies have been restricted to the use of various fatty acids that, although the endogenous ligands, have modest affinity for the receptor. To provide novel, selective pharmacological tools to probe the function of FFA4, we recently developed a highly selective FFA4 agonist, 3-(4-((4-fluoro-4′-methyl-[1,1′-biphenyl]-2-yl)methoxy)phenyl)propanoic acid (TUG-891) (15). Importantly, TUG-891 activates the FFA4 receptor with high potency, efficacy, and selectivity and in a manner similar to that of the natural long chain free fatty acids (16). In this respect, TUG-891 appears to show no stimulus bias and therefore is an excellent tool compound to probe the function of FFA4. Here we used TUG-891 alongside the natural fatty acid α-linolenic acid to explore the potential of FFA4 to be phosphorylated and the consequences of this for interaction of the receptor with arrestin-3 (also designated β-arrestin-2). We demonstrated that human FFA4 was phosphorylated in an agonist-sensitive manner on 5 hydroxyl amino acids located in the C-terminal tail of the receptor. These phosphorylation sites regulated the effectiveness of interaction of the receptor with arrestin-3 but were not the only components of this interaction. In addition, there were structural elements contributed by acidic residues within the C-terminal tail that operated in concert with receptor phosphorylation to allow for full recruitment of arrestin-3. Alteration of these residues or truncation of the full C-terminal tail did not restrict activation of heterotrimeric G proteins. In this way, we made a clear distinction between the mechanism of coupling to heterotrimeric G proteins and arrestin-3 signaling, raising the prospect that ligands can be developed that selectively activate one of these signaling arms over the other. We discuss how such ligands showing stimulus bias might have important clinical benefits.

## EXPERIMENTAL PROCEDURES

### 

#### 

##### Materials

Unless otherwise stated all biochemicals and reagents were from Sigma-Aldrich. TUG-891 was synthesized as described previously (15).

##### Plasmids and Mutagenesis

Plasmids encoding human FFA4 (short isoform) fused at its C terminus to enhanced yellow fluorescent protein (eYFP) and incorporating an N-terminal FLAG epitope and arrestin-3 fused to *Renilla* luciferase (Rluc) were as described previously (15). To generate a C-terminal HA epitope-tagged form of FFA4, the receptor coding sequence was amplified by PCR using the forward (5′-TTTTAAGCTTGCCACCATGTCCCCTGAATGCGC-3′) and reverse (5′-TTTTGGATCCTTAAGCGTAATCTGGAACATCGTATGGGTAGCCAGAAATAATCGACAAGTCA-3′) primers, which incorporated the HA tag sequence followed by a stop codon immediately following the final codon of FFA4. This PCR product was then inserted into the HindIII and BamHI sites of pcDNA5 FRT/TO. To generate FFA4 truncations, the FLAG-FFA4 sequence was amplified from the FLAG-FFA4-eYFP plasmid using the forward primer 5′-TGCTAAGCTTCTTGCCACCATGGACTA-3′ combined with a reverse primer for each truncation as follows: 336, 5′-AAAGGTACCGCAGCAAAAAATTTTCTTCCA-3′; 340, 5′-TTTTGGTACCTGGGAACCAGAAGCAGCA-3′; 345, 5′-AAAAGGTACCAATGGCTCCCTTTTCTGGG-3′; 350,5′-AAAGGTACCAGATGTGTCTGTTAAAATGGC-3′; and 355, 5′-AAAGGTACCGTCATTTCTTTTGACAGATGT-3′. Each PCR product was then inserted into the pcDNA5 FRT/TO vector immediately before the coding sequence for eYFP. Mutations to the FFA4 sequence were incorporated using the QuikChange method (Stratagene, Cheshire, UK), and the identities of all plasmids generated were confirmed through sequencing.

##### Stable Cell Lines

Stable inducible Flp-In^TM^ T-REx^TM^ cells were generated by co-transfecting FLAG-FFA4-eYFP, FLAG-FFA4-TSS/AAA-eYFP, or FLAG-FFA4-340-eYFP with the pOG44 plasmid into parental Flp-In T-REx cells (Life Technologies). Following transfection, hygromycin B was added to the culture medium allowing for polyclonal selection of stable cell lines that inducibly expressed the receptor of interest in response to the antibiotic doxycycline.

Chinese hamster ovary (CHO) cells that stably and constitutively expressed the C-terminal HA epitope-tagged FFA4 or FFA4 containing mutations within the C-terminal tail were generated using the Flp-In system. CHO Flp-In cells were co-transfected with pcDNA5FRT containing FFA4 and pOG44, transfected cells were selected with hygromycin B, and expression of FFA4 was confirmed by immunoblotting with anti-HA antibodies.

##### [^32^P]Orthophosphate Labeling and FFA4 Immunoprecipitation

Cells were plated in 6-well plates at 200,000 cells/well 24 h before experimentation. For phosphorylation experiments, cells were washed three times with Krebs/HEPES buffer without phosphate (118 mm NaCl, 1.3 mm CaCl_2_, 4.3 mm KCl, 1.17 MgSO_4_, 4.17 mm NaHCO_3_, 11.7 mm glucose, 10 mm HEPES (pH 7.4)) and incubated in this buffer containing 100 μCi/ml [^32^P]orthophosphate for 1 h at 37 °C. Cells were stimulated for 5 min with test compounds and immediately lysed by addition of buffer containing 20 mm Tris (pH 7.4), 150 mm NaCl, 3 mm EDTA, 1% Nonidet P-40, 0.5% sodium deoxycholate. FFA4 was immunoprecipitated from the cleared lysates using anti-HA affinity matrix (Roche Applied Science). The washed immunoprecipitates were separated by SDS-PAGE on 10% gels that were dried, and radioactive bands were revealed using autoradiography film. The films were scanned, and bands were quantified using AlphaImager software (Alpha Innotech, San Leandro, CA).

##### FFA4 Purification and Mass Spectrometry

Stably transfected CHO cells expressing FFA4 HA-tagged at the C terminus were grown until confluent in expanded surface rolling bottles at 0.25 rpm in a humidified CO_2_ incubator. For receptor purification, cells from four rolling bottles were harvested, resuspended in 40 ml of Krebs/HEPES buffer, and stimulated with TUG-891 (10 μm) for 5 min. Membranes were then prepared and solubilized by addition of 5 ml of PBS containing 1% Nonidet P-40 plus a mixture of protease and phosphatase inhibitors (Roche Applied Science). After centrifugation at 20,000 × *g*, the resulting supernatant was diluted 1:1 with PBS, and the receptor was then purified on anti-HA affinity matrix. After extensive washing with solubilization buffer containing 0.5% Nonidet P-40, the resin was resuspended in 2× SDS-PAGE sample buffer. The sample was resolved by SDS-PAGE on 10% gels and stained with colloidal Coomassie Blue. Purified FFA4 receptors were excised from the polyacrylamide and washed three times for 15 min with 100 mm triethylammonium bicarbonate (TEAB). Reduction and alkylation of cysteines were performed by addition of 10 mm dithiothreitol in 50 mm TEAB at 60 °C for 30 min followed by addition of 100 mm iodoacetamide in 50 mm TEAB for 30 min in the dark. Gel slices were washed three times for 5 min with 50 mm TEAB containing 50% acetonitrile, finally resuspended in TEAB containing 10% acetonitrile, and incubated overnight at 37 °C with 1 μg of sequencing grade trypsin (Promega, Southampton, UK).

The resulting tryptic peptides were dried and resuspended in 1 ml of buffer containing 250 mm acetic acid and 30% acetonitrile, and phosphorylated peptides were enriched by addition of 20 μl of PHOS-Select^TM^ iron affinity resin and incubation at room temperature for 2 h with mixing. After washing resin twice with loading buffer and once with water, tryptic phosphopeptides were eluted by addition of 200 μl of buffer containing 400 mm ammonium hydroxide and 30% acetonitrile.

LC-MS/MS was carried out upon each sample using an LTQ Orbitrap mass spectrometer (Thermo Fisher Scientific, Rockford, IL). Peptides resulting from in-gel digestion were loaded at high flow rate onto a reverse-phase trapping column (0.3-mm inner diameter × 1 mm) containing 5-μm C_18_ 300-Å Acclaim PepMap medium (Dionex, UK) and eluted through a reverse-phase capillary column (75-μm inner diameter × 150 mm) containing Symmetry C_18_ 100-Å medium (Waters, Elsetree, UK) that was self-packed using a high pressure packing device (Proxeon Biosystems, Odense, Denmark).

The resulting spectra were searched against the UniProtKB/Swiss-Prot database using Mascot (Matrix Science Ltd.) software with peptide tolerance set to 5 ppm and the MS/MS tolerance set to 0.6 Da. Fixed modifications were set as carbamidomethylcysteine with variable modifications of phosphoserine, phosphothreonine, phosphotyrosine, and oxidized methionine. The enzyme was set to trypsin/proline, and up to two missed cleavages were allowed. Peptides with a Mascot score greater than 20 and for which the probability *p* that the observed match was a random event was <0.05 were included in the analysis. The spectra of peptides reported as being phosphorylated were interrogated manually to confirm the precise sites of phosphorylation.

##### Generation of Glutathione S-Transferase (GST) Fusion Constructs

Human FFA4 C-terminal residues Asn^317^–Gly^361^ were inserted into pLEIS 50 to produce C-terminal GST fusions. *Escherichia coli* BL21(DE3) IRL transformed with the fusion constructs or pGEX-2t alone were grown in LB medium containing 50 μg/ml ampicillin, 50 μg/ml chloramphenicol, and 1% (w/v) glucose, and protein expression was induced by addition of isopropyl 1-thio-β-d-galactopyranoside to a final concentration of 100 μm.

##### Generation of Phosphorylation-specific FFA4 Antiserum

A phosphorylation-specific antiserum was raised against the peptide KGAILT_(P)_DTS_(P)_VKR, which corresponds to amino acids 342–353 of human FFA4 in which threonine 347 and serine 350 were phosphorylated. The 87-day program, which included four immunizations, was performed by Eurogentec (Leige Science Park, Seraing, Belgium). The resulting antiserum was purified against the immunizing peptide.

##### Immunocytochemistry

CHO cells expressing FFA4 HA-tagged at the C terminus were seeded onto 20-mm glass coverslips for 24 h prior to experimentation. Cells were washed and incubated for 1 h in Krebs/HEPES buffer prior to treatment. Cells were fixed in PBS containing 4% paraformaldehyde and 0.1% glutaraldehyde for 30 min at room temperature. Anti-HA antibody was used at 5 ng/ml followed by Alexa Fluor^TM^ 546 goat anti-rat secondary antibody at 1:1000 dilution. Data were acquired using an LSM 510 laser-scanning confocal microscope (Zeiss).

##### FFA4/Arrestin-3 Interaction Assay

To assess arrestin-3 recruitment to FFA4, a bioluminescence resonance energy transfer (BRET) assay was used based on our protocol described previously (15). Briefly, HEK293T cells were co-transfected with arrestin-3-Rluc and FLAG-FFA4-eYFP plasmids in a 1:4 ratio using polyethyleneimine. After 24-h incubation, cells were subcultured into poly-d-lysine-coated white 96-well microplates and incubated for a further 24 h prior to the assay. Cells were then washed with Hanks' balanced salt solution and incubated in Hanks' balanced salt solution for 30 min prior to conducting the assay. To initiate the assay, the Rluc substrate coelenterazine h was added to a final concentration of 2.5 μm and incubated for 10 min at 37 °C before the test compound was added. Following a further 5-min incubation, luminescence emissions at 535 and 475 nm were measured using a PHERAstar FS (BMG Labtech, Offenburg, Germany), and the BRET signal was represented as the 535/475 ratio multiplied by 1000 to yield the arbitrary milli-BRET units. For all mutant forms of FFA4, the BRET signals obtained are expressed as a percentage of the maximum milli-BRET units obtained for TUG-891 at the wild type receptor. To conduct kinetic arrestin-3 recruitment experiments, cells were incubated at 37 °C in the PHERAstar FS after coelenterazine h had been added, and BRET measurements were taken at 6-s intervals. In these experiments, test compound was added using PHERAstar FS injectors, allowing for continuous measurement during and immediately following compound addition.

##### Mobilization of Intracellular Ca^2+^

Intracellular Ca^2+^ measurements were taken from Flp-In T-REx 293 cells induced to express the receptor of interest by treatment with 100 ng/ml doxycycline according to a protocol described previously (16). All data are represented as a percentage of the maximum response obtained for TUG-891 within the same cell line.

##### High Content Imaging Quantitative Internalization Assay

Quantitative measurements of FFA4 internalization in Flp-In T-REx 293 cells induced to express the receptor of interest with 100 ng/ml doxycycline were taken using a high content imaging system following our protocol described previously (16). For these experiments, quantification was based on the intensity of eYFP fluorescence observed within internalized spots while cell number was controlled for using a Hoechst nuclear stain. As the signal measured in this assay system is strongly dependent on receptor expression level, estimates comparing the efficacy of internalization between different cell lines were not made. Therefore data are represented as a percentage of the maximum response obtained for TUG-891 within the same cell line except for the FFA4-340 truncation, which is expressed as a percentage of the response in the wild type cells as TUG-891 had no measurable effect on this receptor variant.

##### Data Analysis and Curve Fitting

All data presented represent means ± S.E. of at least three independent experiments. Data analysis and curve fitting were carried out using the GraphPad Prism software package. Concentration-response data were plotted on a log axis where the untreated vehicle control condition was plotted at 1 log unit lower than the lowest test concentration of ligand and fitted to three-parameter sigmoidal concentration-response curves. Statistical analyses were carried out using standard *t* tests, one-way analysis of variance with Tukey's post hoc analysis, or two-way analysis of variance combined with Bonferroni post hoc analysis as appropriate.

## RESULTS

### Agonist-promoted Phosphorylation of FFA4

**FIGURE 1.**
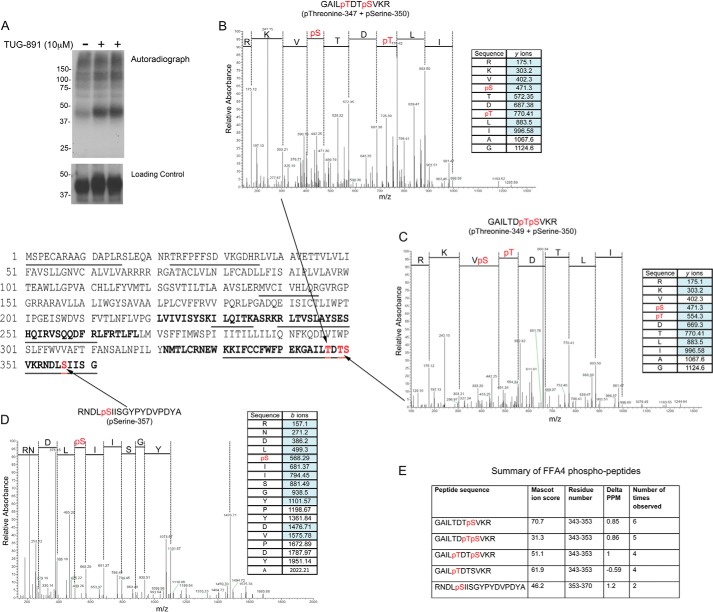
**Mass spectrometry identifies four distinct sites of phosphorylation in FFA4.** CHO cells stably expressing C-terminally HA-tagged FFA4 were either labeled with ^32^P (*A*) or used to immunoprecipitate and then digest the receptor for analysis using mass spectrometry (*B–E*). In the ^32^P labeling studies, cells were treated with the agonist TUG-891 (10 μm) or vehicle for 5 min prior to sample preparation. An autoradiogram (*upper panel*) and loading control representing an anti-HA immunoblot of the same samples (*lower panel*) are shown from a representative experiment. *B–D*, representative mass spectra and associated fragmentation tables are shown that cover the four phosphorylated residue, Thr^347^, Thr^349^, Ser^350^, and Ser^357^ identified in various experiments (noted in *bold* are the 3rd intracellular loop and C-terminal tail within the primary sequence of FFA4, *underlined* sections represent FFA4 peptides observed in mass spectrometry experiments and in *red* are amino acids identified as being phosphorylated). *E*, a summary of the overall data set.

We have previously described key signaling responses of the FFA4 receptor following stimulation with the natural fatty acid agonist α-linolenic acid and the FFA4-selective chemical ligand TUG-891 (16). These included enhanced phosphorylation of the receptor as monitored by increased incorporation of ^32^P into a polypeptide with an apparent molecular mass between 45 and 50 kDa in CHO cells stably expressing HA-tagged human FFA4 (Fig. 1*A*). Enhanced incorporation of ^32^P into polypeptide(s) with an apparent molecular mass of some 100 kDa was also observed (Fig. 1*A*). These may represent either dimeric complexes of FFA4 or the monomeric receptor in complex with other protein(s) of similar molecular mass.

**FIGURE 2.**
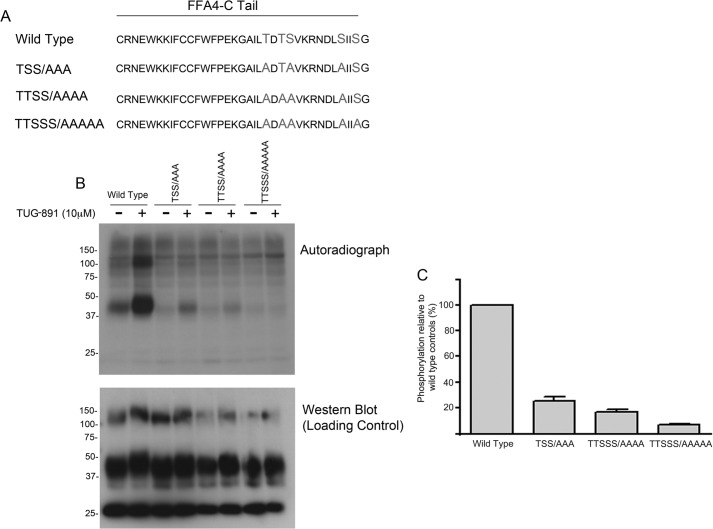
**Mutational analysis of FFA4 confirms these four sites of agonist-induced phosphorylation and identifies a further site.** ^32^P labeling studies of FFA4 were performed as detailed in Fig. 1 using CHO cells stably expressing either wild type HA-tagged FFA4 or the various mutants illustrated in *A. B*, an autoradiogram (*upper panel*) and loading control representing an anti-HA immunoblot of the same samples (*lower panel*) from a representative experiment. TUG-891 was added where specified for 5 min. *C*, quantification of studies akin to those shown in *B* (mean ± S.E.; *n* = 3). *Error bars* represent S.E.

To investigate the details of ligand-promoted phosphorylation of this receptor, we conducted a mass spectrometry-based proteomics study to determine the precise sites of phosphorylation within FFA4. In these experiments, HA-tagged FFA4 was purified following stimulation with TUG-891 (10 μm; 5 min). Mass spectrometry-based proteomic analysis of tryptic peptides generated from the isolated FFA4 revealed excellent peptide coverage of the third intracellular loop and C-terminal tail (Fig. 1). Importantly, this included peptides that contained 11 of the 16 serine and threonine residues within these intracellular domains. MS/MS sequencing revealed four phosphorylation sites (Thr^347^, Thr^349^, Ser^350^, and Ser^357^), all of which were located in the C-terminal tail (Fig. 1, *B–E*). Recombinant CHO cells were generated that stably expressed mutant FFA4 receptors in which these phosphorylation sites were mutated to alanine residues. We initially mutated Thr^347^, Ser^350^, and Ser^357^ to alanine in a mutant designated TSS/AAA (Fig. 2*A*). Phosphorylation of this mutant in response to TUG-891 was reduced by 74.4 ± 3.0% (*n* = 3) (Fig. 2, *B* and *C*). Further mutation of Thr^349^ to generate the mutant TTSS/AAAA removed all the phosphorylation sites identified by mass spectrometry (Fig. 2*A*). This mutant still showed some agonist-regulated phosphorylation but markedly less than the TSS/AAA mutant (Fig. 2, *B* and *C*). The only other possible serine/threonine phosphorylation site in the C-terminal tail of FFA4 was Ser^360^. This residue was mutated in combination with Thr^347^, Thr^349^, Ser^350^, and Ser^357^ to generate the mutant TTSSS/AAAAA (Fig. 2*A*). No evidence of agonist-regulated phosphorylation was seen for this mutant (Fig. 2, *B* and *C*), indicating that the sites of agonist-regulated phosphorylation in FFA4 were Thr^347^, Thr^349^, Ser^350^ Ser^357^, and Ser^360^.

### Characterization of Phosphospecific Antibodies

**FIGURE 3.**
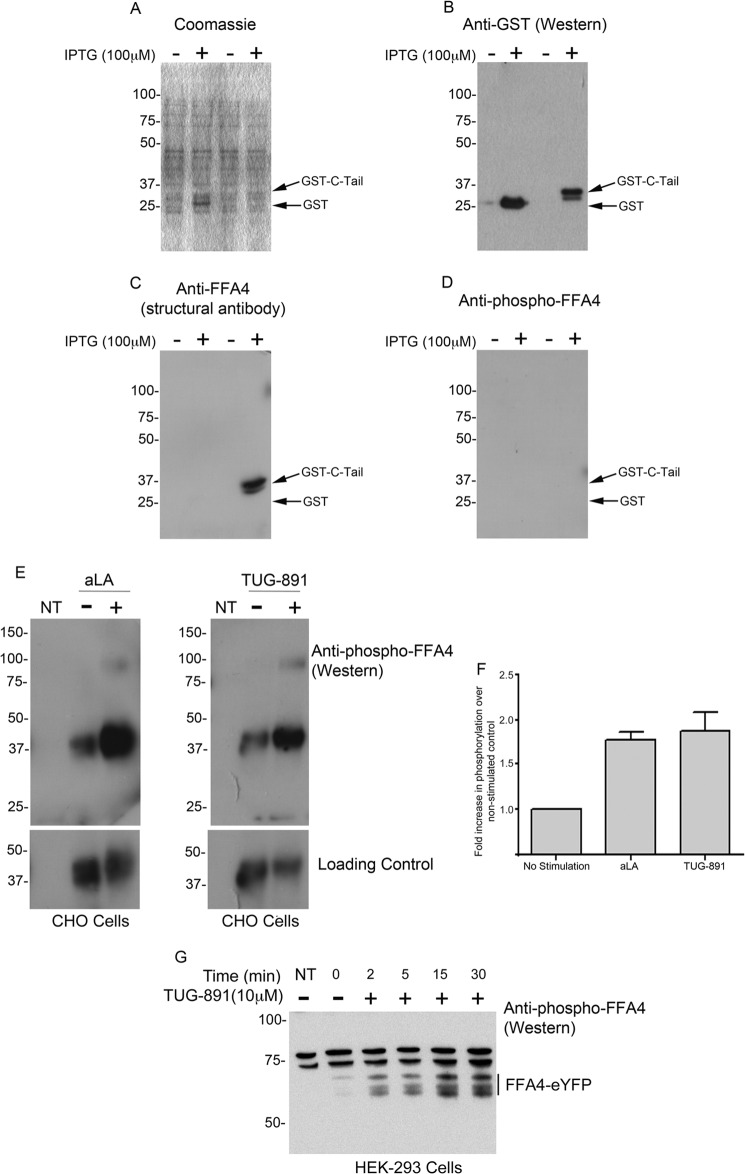
**Characterization and use of an FFA4 phosphospecific antiserum.** An antiserum was raised to identify Thr(P)^347^,Ser(P)^350^ within the C-terminal tail of human FFA4 (see “Experimental Procedures” for details). A GST fusion protein containing the final 45 amino acids of the C-terminal tail of FFA4 was generated and expressed in *E. coli* in a manner dependent upon addition of isopropyl 1-thio-β-d-galactopyranoside (*IPTG*). Coomassie Blue stain (*A*) and an anti-GST immunoblot (*B*) of SDS-PAGE-resolved lysates of such bacteria are shown. Following chromatography over a column containing the Thr(P)^347^,Ser(P)^350^ peptide antigen, serum that was not retained (potential anti-FFA4 non-phosphostate-dependent structural antibodies) was separated from antibodies that were retained (potential anti-FFA4 phosphospecific antibodies). These (*C*, structural; *D*, phosphospecific) were used in immunoblots of the samples described in *A* and *B*. Lysates of non-transfected (*NT*) CHO cells and those expressing FFA4-HA that were exposed to α-linolenic acid (*aLA*) or TUG-891 as indicated were resolved and immunoblotted with the phosphospecific antibodies (*E*, *upper panel*). Loading controls (*E*, *lower panel*) represent the same samples immunoblotted to detect the HA tag. Such immunoblots were quantified (*F*). Data represent quantification of experiments shown in *E* (mean ± S.E.; *n* = 3). *G*, HEK293T cells were transiently transfected to express FFA4-eYFP and subsequently treated for the indicated times with TUG-891 (10 μm). Membrane preparations were resolved by SDS-PAGE and immunoblotted with the phosphospecific anti-FFA4 antibodies. *Error bars* represent S.E.

Phosphospecific antibodies were raised to a doubly phosphorylated peptide containing phosphates on residues corresponding to Thr^347^ and Ser^350^ of FFA4. This phosphopeptide was chosen because there was good evidence from the mass spectrometry studies that these residues were phosphorylated simultaneously and because this doubly phosphorylated peptide was predicted to be highly immunogenic. The antiserum generated from the immunization was subjected to affinity purification that resolved phosphospecific antibodies from non-phosphospecific antibodies (described herein as “structural” antibodies). To test the specificity of the antibodies, a GST bacterial fusion protein containing the final 45 amino acids of the C-terminal tail of FFA4 was prepared. Because bacteria do not generally phosphorylate mammalian fusion proteins, the FFA4-C-tail fusion protein acted as a negative control for the phosphospecific antibodies in immunoblots of bacterial lysates containing this protein (Fig. 3*A*). The FFA4-C-tail fusion protein was readily identified in immunoblots using a commercial antibody against GST (Fig. 3*B*) and by the structural FFA4 antibodies (Fig. 3*C*) but was not detected with the putative phosphospecific antibody fraction (Fig. 3*D*). However, the phosphospecific FFA4 antibody fraction readily detected FFA4 expressed in CHO cells in a manner that was up-regulated by ∼1.8-fold in response to acute treatment with either α-linolenic acid or TUG-891 (Fig. 3, *E* and *F*). Importantly, the band corresponding to phosphorylated FFA4 in immunoblots of transfected cell extracts was absent in lysates prepared from control non-transfected CHO cells (Fig. 3*E*). The phosphospecific FFA4 antibodies also effectively identified a C-terminally eYFP-tagged form of FFA4 transiently expressed in HEK293T cells with, once again, marked increases in detection of the receptor following treatment of the cells with TUG-891 for various times (Fig. 3*G*), indicating that FFA4 phosphorylation appears to operate in a similar manner in both CHO cells and HEK293 cells.

### Agonist-regulated Phosphorylation of FFA4 Is Not via Protein Kinase C (PKC)

**FIGURE 4.**
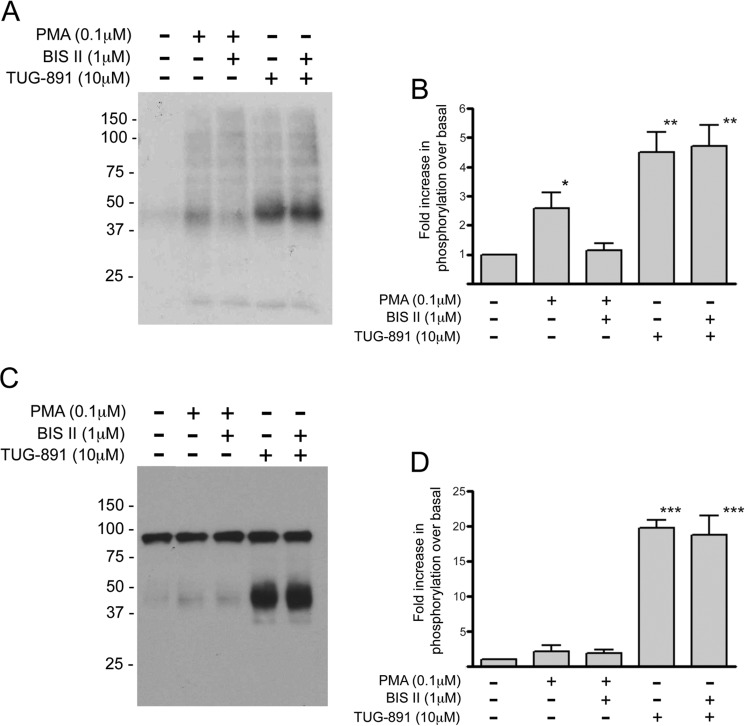
**PKC can phosphorylate FFA4 but does not contribute to TUG-891-mediated phosphorylation of Thr(P)^347^ and Ser(P)^350^.** CHO cells as in Fig. 3*E* were used in ^32^P labeling experiments as detailed in Fig. 1. Cells were exposed to phorbol 12-myristate 13-acetate (*PMA*), bisindolylmaleimide II (*BIS II*), TUG-891, or combinations of these as detailed. Subsequently, cell lysates were subjected to immunoprecipitation with anti-HA antibodies and resolved by SDS-PAGE, and autoradiography was performed. *A* represents a representative autoradiogram, and *B* represents quantification of incorporation of ^32^P into FFA4 in such experiments. *C* and *D* are equivalent experiments in which the cell lysates were probed with the dual Thr(P)^347^,Ser(P)^350^ phosphospecific antibodies (data in *B* and *D* represent means ± S.E.; *n* = 3). *Error bars* represent S.E. **, *p* < 0.01; ***, *p* < 0.001.

To investigate the possibility that PKC isoforms might mediate agonist-regulated FFA4 phosphorylation, experiments were performed using ^32^P labeling and immunoblotting with the phosphospecific antibodies. Treatment of cells with the phorbol ester phorbol 12-myristate 13-acetate for 10 min resulted in global phosphorylation of FFA4 that was significantly greater than basal (Fig. 4, *A* and *B*). This suggested that PKC can mediate heterologous phosphorylation, consistent with previous observations (17, 20). However, agonist-dependent phosphorylation was not PKC-mediated because pretreatment of cells with bisindolylmaleimide II, a PKC inhibitor, for 10 min did not decrease the levels of agonist (TUG-891)-dependent phosphorylation (Fig. 4, *A* and *B*). Importantly, in control experiments, bisindolylmaleimide II pretreatment did inhibit phorbol 12-myristate 13-acetate-mediated phosphorylation (Fig. 4, *A* and *B*). Interestingly, the cells were serum-starved overnight in these experiments, which appeared to reduce the basal phosphorylation. For this reason, the -fold increase in phosphorylation observed in this series of experiments is higher than reported for our other experiments (*e.g.* in Figs. 2 and 3).

In contrast to a previous study (20), PKC did not appear to mediate phosphorylation of Thr^347^ and Ser^350^ when tested in immunoblot experiments using the phosphospecific antibodies recognizing these sites (Fig. 4, *C* and *D*). These experiments were carried out again on serum-starved cells and with a 1 μm phorbol 12-myristate 13-acetate concentration equivalent to that used by Burns *et al.* (20). Thus, our studies demonstrate that, although PKC has the potential to phosphorylate FFA4 and that FFA4 is G_q/11_-coupled and therefore able to signal through PKC, there does not appear to be a feedback loop where PKC can further phosphorylate the receptor following agonist stimulation.

### Arrestin-3 Interaction with the C-terminal Tail of FFA4

**FIGURE 5.**
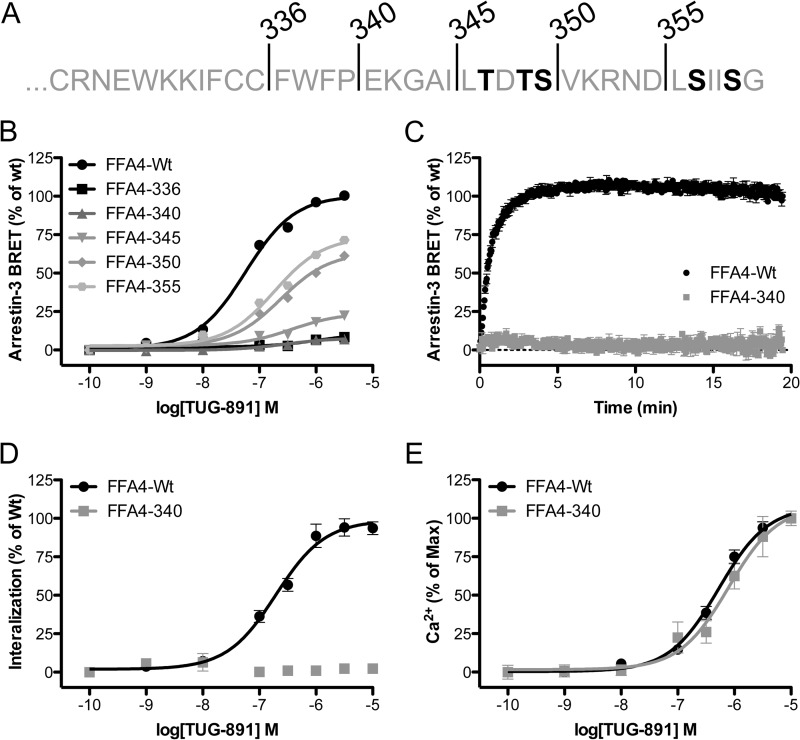
**Truncation of elements of the C-terminal tail of FFA4 limits agonist-induced interaction with arrestin-3 but not Ca^2+^ signaling potency.** A series of truncation constructs of FFA4 were generated and linked in-frame to eYFP. *A*, *numbers* and *lines* highlight the final amino acid in each truncation, and the sites of agonist-mediated phosphorylation identified directly by mass spectrometry (Fig. 1) are *bold. B*, wild type FFA4-eYFP and each of the truncations were then used in BRET-based arrestin-3 interaction assays after transient co-expression in HEK293T cells with Rluc-tagged arrestin-3. Concentration-response curves for TUG-891 to promote FFA4/arrestin-3 interaction at each truncation are displayed. *C*, extending the measurement time to 20 min (10 μm TUG-891) indicates that the FFA4-340 truncation still does not interact with arrestin-3. *D*, normalized high content imaging internalization measurements in response to TUG-891 suggest that FFA4-340 does not internalize. Data are shown for FFA4-eYFP and FFA4-340-eYFP expressed stably in Flp-In T-REx 293 cells. Wild type FFA4-eYFP yielded a raw signal window between 99 ± 9 arbitrary fluorescence units/cell in unstimulated cells and 603 ± 44 arbitrary fluorescence units/cell in TUG-891-treated cells, whereas FFA4-340 yielded values of 38 ± 3 and 44 ± 3 arbitrary fluorescence units/cell for untreated and TUG-891-treated cells respectively. *E*, the potency of TUG-891 to elevate [Ca^2+^]*_i_* in acute signaling studies is unaffected by the FFA4-340 C-terminal tail truncation. These experiments yielded raw Fura 2 ratio signals of 0.03 ± 0.02 and 1.52 ± 0.08 for basal and TUG-891 (10 μm)-stimulated wild type FFA4, respectively, and values of 0.09 ± 0.02 and 0.88 ± 0.02 for basal and TUG-891 (10 μm)-stimulated FFA4-340, respectively. *Error bars* represent S.E.

FFA4 interacts effectively with arrestin-3 in an agonist-dependent manner (16). To begin to dissect the potential role of the C-terminal tail of FFA4 in the regulation of receptor signaling, we truncated the receptor following residue 336 immediately after the pair of Cys residues that are likely to be palmitoylated and define the end of the putative intracellular helix 8. In addition, we generated a series of less extensive truncations, the first of which after Asp^355^, removing the final 6 amino acids of FFA4, and then in 5-amino acid blocks up to Pro^340^ (Fig. 5*A*). When tested in a BRET-based arrestin-3 interaction assay, 5 min of TUG-891 (10 μm) treatment yielded a strong increase in arrestin-3-Rluc/FFA4-eYFP BRET, increasing the basal signal of 63 ± 6 milli-BRET units to the TUG-891-stimulated signal of 399 ± 19 milli-BRET units. Extending these studies to also test the truncations demonstrated that these forms produced clear reductions in BRET signal that correlated with the extent of FFA4 truncation (Fig. 5*B*). Specifically, although signals for 336 and 340 truncations were all but absent, those to residues 345, 350, and 355 showed increasing BRET response efficacies that were measured to be 25 ± 2, 64 ± 4, and 73 ± 4% of the wild type FFA4 signal, respectively.

To explore the contribution of the FFA4 C-terminal tail in more detail, we chose to focus on the FFA4-340 variant as the least extensive truncation for which interaction with arrestin-3 was effectively eliminated. To first confirm that arrestin-3 interaction was eliminated in this mutant, kinetic BRET studies were carried out and demonstrated that even with up to a 20-min treatment TUG-891 was unable to stimulate arrestin-3 interaction with FFA4-340 (Fig. 5*C*). This lack of agonist-induced interaction with arrestin-3 also correlated with a lack of agonist-induced internalization of the truncated FFA4-340 mutant (Fig. 5*D*). Although these data demonstrate the essential nature of the C-terminal tail of FFA4 for arrestin-3 interaction and internalization, a concentration-dependent (pEC_50_ = 6.10 ± 0.13) calcium mobilization in response to TUG-891 with similar potency to wild type FFA4 (pEC_50_ = 6.28 ± 0.05) was maintained for the FFA4-340 C-terminal tail truncation (Fig. 5*E*), indicating that the distal C terminus beyond the putative intracellular helix 8 is not required for acute G_q/11_ coupling of FFA4. Thus, FFA4 displayed classical GPCR bimodal signaling properties where one arm consists of agonist-mediated interaction with arrestins in a process that drives arrestin-dependent mechanisms, including receptor internalization, and the other arm independently couples the receptor to heterotrimeric G proteins and G protein-mediated signaling pathways (*e.g.* calcium mobilization) (18).

### Structural and Phosphorylation-dependent Elements Combine to Regulate FFA4/Arrestin-3 Interaction

#### 

##### Phosphorylation-dependent Arrestin-3 Interaction

**FIGURE 6.**
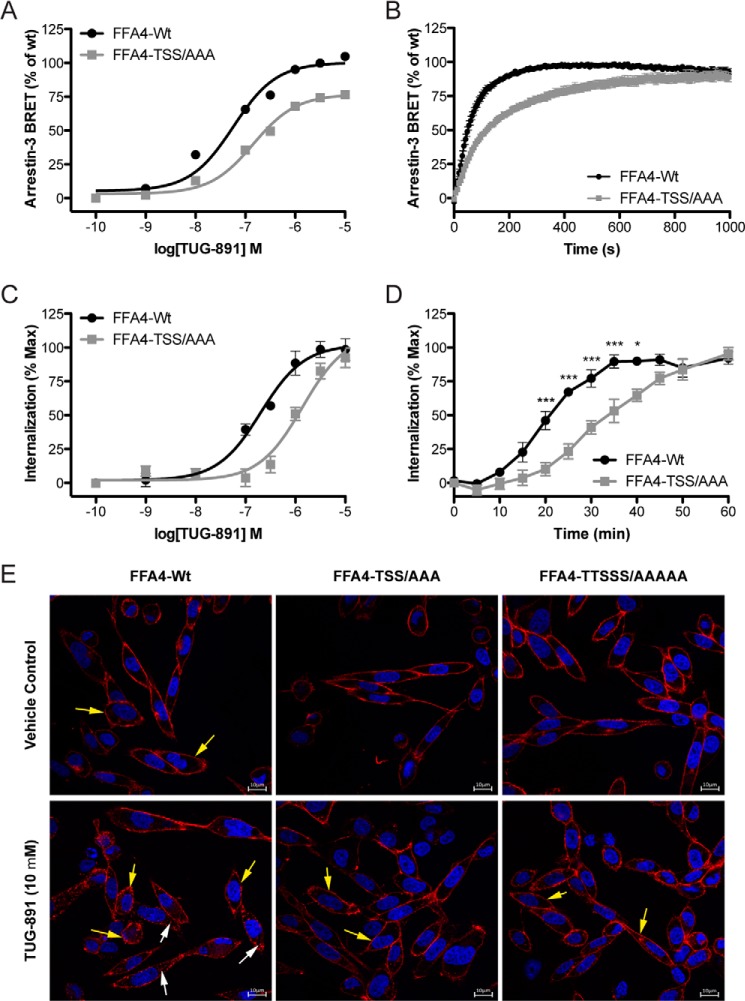
**Mutation of key sites of agonist-regulated phosphorylation reduces the potency and efficacy of TUG-891 to promote interactions with arrestin-3 and slows the kinetic of the process.** *A*, comparison of arrestin-3 interaction TUG-891 concentration responses for wild type FFA4 and the TSS/AAA mutant at a 5-min measurement time point. *B*, the kinetics of FFA4/arrestin-3 interaction are compared with those of the TSS/AAA mutant. *C*, TUG-891 concentration responses for FFA4 and FFA4-TSS/AAA internalization measured after 45-min ligand treatment. *D*, the kinetics of FFA4 and FFA4-TSS/AAA internalization are shown following treatment with 10 μm TUG-891. *, *p* < 0.05; ***, *p* < 0.001 compared with wild type response at the same time point. *E*, following stable expression in CHO cells of wild type, TSS/AAA, and TTSSS/AAAAA, HA-tagged FFA4 is present predominantly at the cell surface as detected by anti-HA immunocytochemistry. Following exposure to TUG-891 (10 μm) for 30 min, the extent of internalization of the TSS/AAA and TTSSS/AAAAA mutants is substantially lower than for wild type. *Yellow arrows* illustrate cell surface receptor, and *white arrows* highlight internalized receptor. *Scale bars*, 10 μm. *Error bars* represent S.E.

A number of the C-terminal truncations eliminated amino acids that become phosphorylated in an agonist-dependent manner. To establish the importance of phosphorylation in the interaction of FFA4 with arrestin-3, the FFA4 mutant designated TSS/AAA containing alanine substitutions in three of the phosphorylation sites (Thr^347^, Ser^350^, and Ser^357^), which resulted in a 74% reduction in agonist-mediated phosphorylation (Fig. 2*B*), was used in arrestin-3 recruitment assays. These experiments demonstrated that the mutant receptor showed a significant decrease in both potency (for TUG-891 at wild type FFA4, pEC_50_ = 7.23 ± 0.08, and for FFA4-TSS/AAA, pEC_50_ = 6.85 ± 0.11; *p* < 0.05) and efficacy (77 ± 2% of wild type response; *p* < 0.001) for arrestin-3 recruitment measured after 5 min in the BRET assay (Fig. 6*A*). Extending the time course of measurement demonstrated that the TSS/AAA mutant showed a slowing in the kinetics of association with arrestin-3: wild type FFA4 interacted with arrestin-3 with a *t*_½_ of 46 ± 6 s, and FFA4-TSS/AAA interacted with arrestin-3 with a significantly longer (*p* < 0.001) *t*_½_ of 121 ± 10 s (Fig. 6*B*). This was linked to a disruption of agonist-mediated receptor internalization where FFA4-TSS/AAA showed a significant (*p* < 0.05) decrease in the potency for TUG-891 to stimulate receptor internalization (pEC_50_ = 5.81 ± 0.22 compared with 6.68 ± 0.11 for wild type FFA4) (Fig. 6*C*) and a significantly reduced time course for internalization (Fig. 6*D*). This disruption in receptor internalization was also evident in CHO cells stably expressing forms of HA-tagged FFA4. Here anti-HA immunostaining indicated a predominantly plasma membrane and uniform localization for both wild type FFA4 and FFA4-TSS/AAA (Fig. 6*E*, *yellow arrows*) under basal conditions, whereas following addition of TUG-891, a more punctate membrane distribution and intracellular vesicle-like staining became apparent (Fig. 6*E*, *white arrows*) in cells expressing wild type FFA4 (Fig. 6*E*). These changes in membrane distribution and the appearance of receptor in intracellular vesicles were less evident but not absent in cells expressing the phosphorylation-limited TSS/AAA mutant (Fig. 6*E*).

**FIGURE 7.**
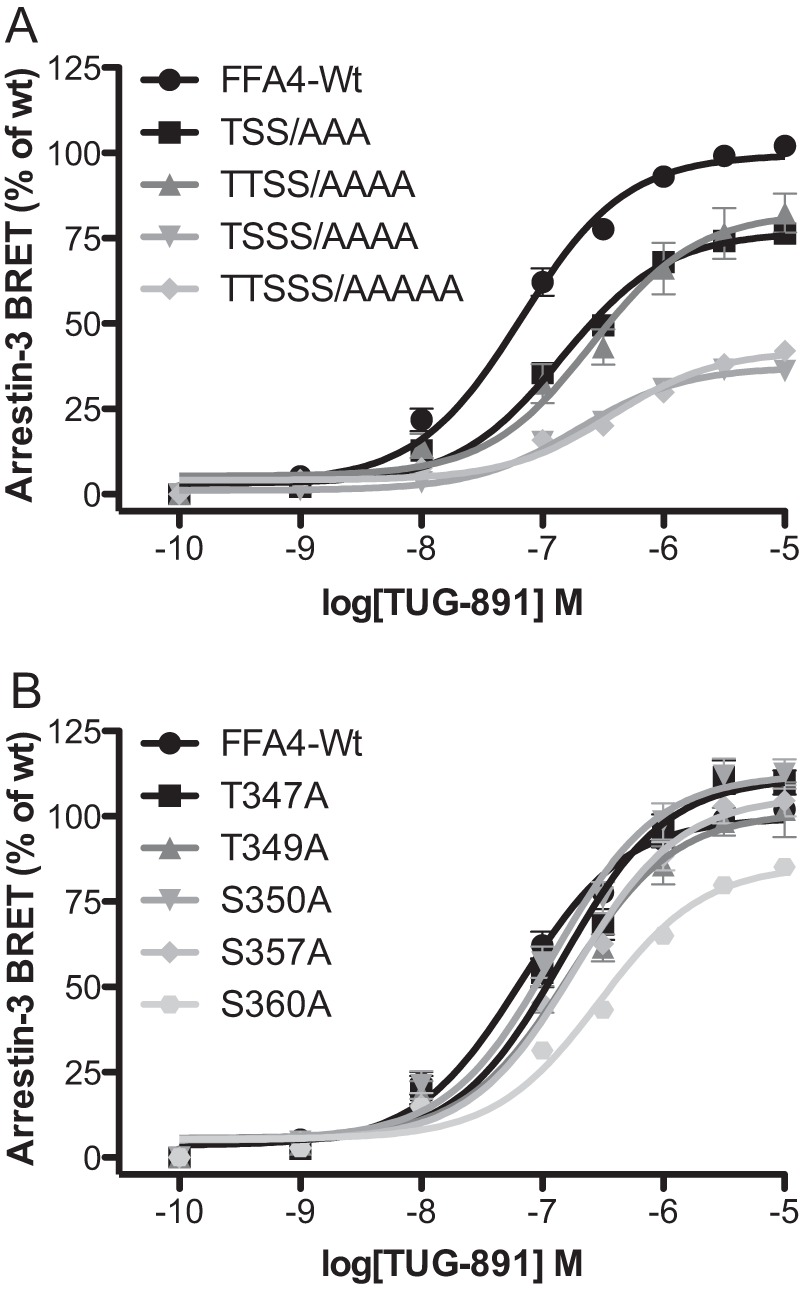
**Hydroxyl amino acids that are targets for TUG-891-regulated phosphorylation play an important but not exclusive role in agonist-induced FFA4/arrestin-3 interactions.** *A*, mutations of FFA4 that eliminate three key sites of agonist-mediated phosphorylation (TSS/AAA) as well as a combination of this with additional Ala mutation of Thr^349^ and Ser^360^ (TTSS/AAAA and TSSS/AAAA, respectively) and a form of the receptor in which all five hydroxyl amino acids were converted to Ala (TTSSS/AAAAAA) were used in BRET-based arrestin-3 interaction studies in response to TUG-891 and compared with wild type FFA4. *B*, the effects of individual mutation of each of these hydroxyl amino acids in this assay. *Error bars* represent S.E.

To further probe the role of phosphorylation, mutations to eliminate the other sites of phosphorylation were made. Interestingly, mutating Thr^349^ in addition to the TSS/AAA mutations (to generate the mutant TTSS/AAAA) did not have a significant further impact on arrestin-3 recruitment efficacy (*E*_max_ = 77 ± 2% for TSS/AAA, and *E*_max_ = 84 ± 7% for TTSS/AAAA) (Fig. 7*A*). This is despite the fact that the TTSS/AAAA mutant showed less phosphorylation in response to agonist than the TSS/AAA mutant (Fig. 2, *B* and *C*). This suggested that it is not necessarily only the bulk negative charge at the C-terminal tail of the receptor resulting from phosphorylation that is important for the arrestin-3 recruitment signal but also the specific location of the negative charge. In support of this notion, adding mutation of Ser^360^ to FFA4-TSS/AAA to generate the mutant TSSS/AAAA did significantly (*p* < 0.001) further reduce arrestin-3 recruitment efficacy (38 ± 2%) (Fig. 7*A*). Finally, combining the Thr^349^ and Ser^360^ mutations to generate the complete phosphorylation-negative TTSSS/AAAAA mutant (Fig. 2*B*) resulted in a BRET response of 42 ± 2%, which was significantly reduced compared with either TSS/AAA or TTSS/AAAA (*p* < 0.001) but was not significantly different from the response to the TSSS/AAAA mutant (Fig. 7*A*). These experiments indicated that phosphorylation sites Thr^347^, Ser^350^, Ser^357^, and Ser^360^ contribute to arrestin-3 recruitment and that Thr^349^ appears to play a less significant role, indicating that there may be some redundancy in this cluster of phosphorylation sites (Fig. 7*A*). Importantly, mutating each site individually had little impact on arrestin-3 recruitment with only the S360A mutant showing a significant (*p* < 0.001) but relatively small reduction in efficacy to 85 ± 1% of wild type FFA4 response (Fig. 7*B*). These data indicate that there is no “master” phosphorylation event and that it is likely an ensemble of phosphorylation events that regulate the phosphorylation-dependent component of FFA4/arrestin-3 interaction and arrestin-3-dependent processes, including receptor internalization.

##### Structural Components Also Regulate FFA4/Arrestin-3 Interaction

The above studies indicated a role for agonist-mediated receptor phosphorylation in arrestin-3 recruitment to FFA4. However, removal of all the sites of phosphorylation (*i.e.* mutant TTSSS/AAAA) did not eliminate arrestin-3 recruitment in response to agonist (Fig. 7*A*). This indicated that factors in addition to receptor phosphorylation must contribute to FFA4/arrestin-3 interaction. To investigate this, additional mutations were generated to define potential structural elements on FFA4 that might regulate arrestin-3 interaction.

**FIGURE 8.**
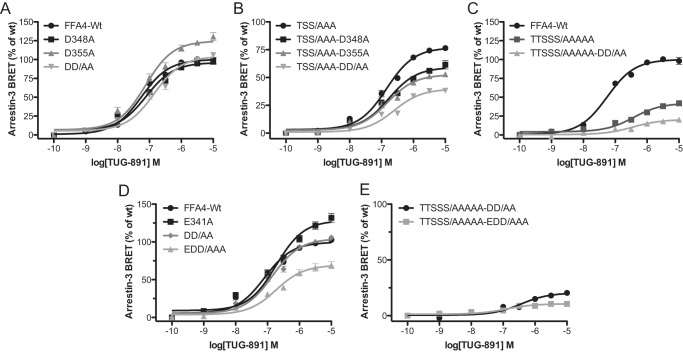
**Negatively charged residues in the C-terminal tail of FFA4 also contribute to the effectiveness of agonist-induced interactions with arrestin-3.** *A*, arrestin-3 recruitment concentration-response curves to TUG-891 obtained from wild type FFA4 containing Ala replacement mutants for Asp^348^ or Asp^355^ as well as a combined DD/AA mutant. *B*, similar experiments using mutants generated from the TSS/AAA phosphodeficient form of FFA4. *C*, arrestin-3 interaction studies performed after replacement of both Asp residues with Ala in the background of the mutant in which all five hydroxyl amino acids had been converted to Ala to generate the TTSSS/AAAAA-DD/AA mutant. *D*, TUG-891 arrestin-3 interaction studies carried out with the E341A mutation incorporated into wild type or DD/AA (to make EDD/AAA) forms of FFA4. *E*, mutation E341A was also carried out in the form of FFA4 with all hydroxyl amino acids and Asp residues converted to Ala (TTSSS/AAAAA-DD/AA) to generate TTSSS/AAAAA-EDD/AAA. The results with this mutant in the arrestin-3 interaction assay are shown. *Error bars* represent S.E.

The initial focus centered on the negative charges contributed by the Asp residues, Asp^348^ and Asp^355^, as Asp residues are often utilized as “phosphomimetics” and might combine with the negative charges produced by phosphorylation of nearby phosphoacceptor sites to further promote arrestin-3 interaction. Indeed, our FFA4 truncation data appeared to support this hypothesis as a significant increase in arrestin-3 interaction efficacy (*p* < 0.05) was observed for FFA4-355 (73 ± 4%) over FFA4-350 (63 ± 4%) despite the fact that this block of amino acids contains no phosphorylation sites but does possess an Asp at position 355 (Fig. 5*B*). To test the role of Asp^348^ and Asp^355^, alanine mutations of these sites individually and in combination (DD/AA) were incorporated into wild type FFA4. When tested in the BRET assay, none of these mutations significantly reduced FFA4/arrestin-3 response efficacy (Fig. 8*A*). However, when these mutations were incorporated into the phosphorylation-deficient FFA4-TSS/AAA, both TSS/AAA-D348A (60 ± 6%; *p* < 0.05) and TSS/AAA-D355A (52 ± 3; *p* < 0.001) significantly reduced efficacy compared with TSS/AAA (77 ± 2%) (Fig. 8*B*). Combining these mutations to make the TSS/AAA-DD/AA mutant resulted in a further reduction in efficacy to 40 ± 1% (Fig. 8*B*). The impact of Asp^348^ and Asp^355^ mutation was also apparent in the complete phosphorylation-negative mutant because removing these aspartate residues in the FFA4-TTSSS/AAAAA mutant to generate the TTSSS/AAAAA-DD/AA mutant also significantly (*p* < 0.001) reduced the efficacy of interaction with arrrestin-3 (*E*_max_ = 42 ± 2% for TTSSS/AAAAA, and *E*_max_ = 21 ± 1% for TTSSS/AAAAA-DD/AA) (Fig. 8*C*).

The only other source of negative charge in the distal portion of the C-terminal tail of FFA4, demonstrated above to be essential for arrestin-3 recruitment (Fig. 5*A*), is the Glu residue Glu^341^. A gain of arrestin-3 interaction for the FFA4-345 truncation compared with the inactive FFA4-340 truncation suggested some role for residues 341–345. Interestingly, although the E341A mutation alone did not significantly reduce arrestin-3 interaction compared with wild type, mutation of this residue in addition to Asp^348^ and Asp^355^ to generate an EDD/AAA mutant possessing no permanent negative charges did result in a significant (*p* < 0.001) reduction in arrestin-3 efficacy (69 ± 7% of wild type) (Fig. 8*D*). Thus, in combination, the three acidic residues in the C-terminal tail of FFA4 provide structural components that contribute to the effectiveness of arrestin-3 recruitment.

Finally, having demonstrated that Glu^341^ does appear to contribute to FFA4/arrestin-3 interaction, we generated a further mutant where E341A was incorporated into the TTSSS/AAAA-DD/AA mutant to generate the TTSSS/AAAAA-EDD/AAA form of FFA4 lacking all possible phosphorylation sites and structural negative charges. This mutant resulted in a further significant (*p* < 0.001) reduction in efficacy from 21 ± 1% for TTSSS/AAAAA-DD/AA to only 11 ± 3% for TTSSS/AAAAA-EDD/AAA (Fig. 8*E*).

##### Summary: Arrestin-3 Interaction Relies on Both Phosphorylation and Structural Components

**FIGURE 9.**
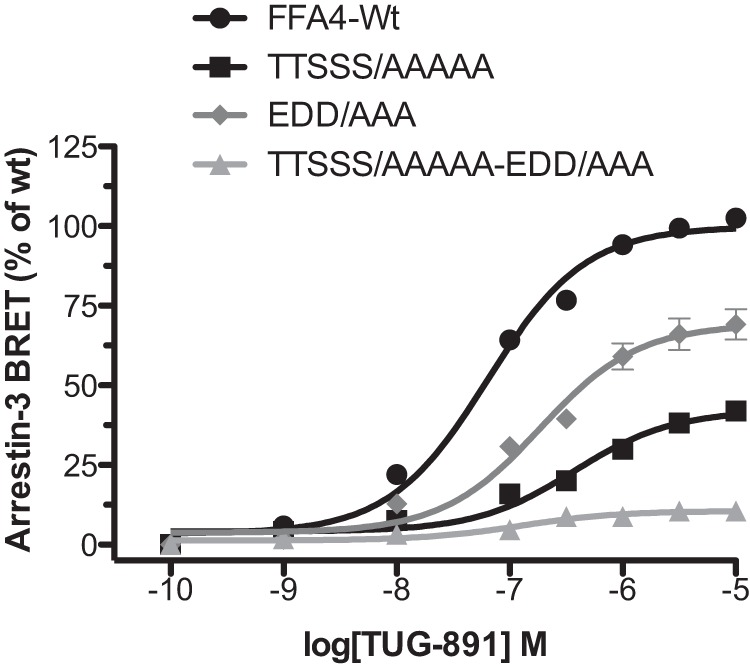
**Both structural elements and receptor phosphorylation determine arrestin-3 interaction with the free fatty acid receptor FFA4.** A synopsis of the key observations that both residues with fixed negative charge and hydroxyl amino acids that become phosphorylated in response to receptor activation contribute to the efficacy of TUG-891-induced interactions between FFA4 and arrestin-3. The TTSSS/AAAAA-EDD/AAA mutant is virtually uncoupled from interacting with arrestin-3. *Error bars* represent S.E.

**FIGURE 10.**
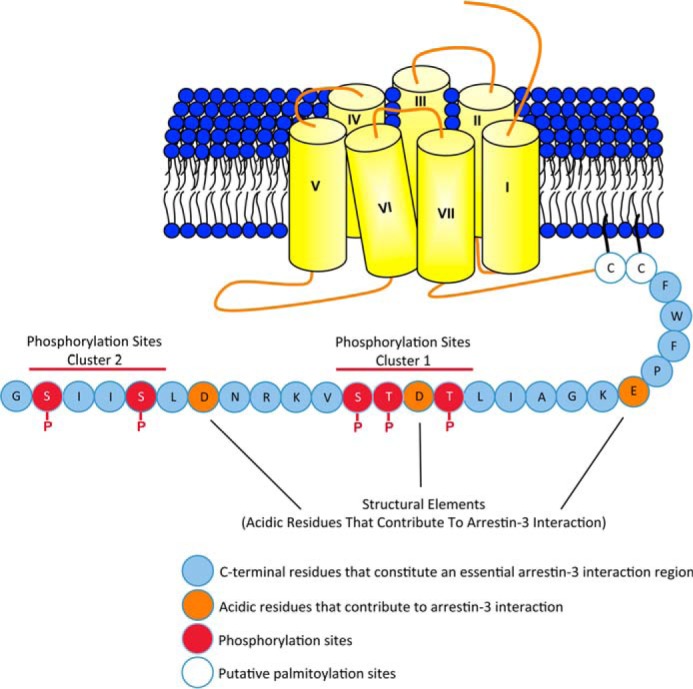
**A schematic summarizing the contributions made by receptor phosphorylation and structural elements within the C-terminal tail to the recruitment of arrestin-3 to FFA4.** We demonstrate here that the residues within the C-terminal tail of FFA4 downstream of the putative palmitoylated pair of cysteine residues are essential for the agonist-mediated interaction of FFA4 with arrestin-3. Furthermore, using mass spectrometry and mutagenesis, it was established that there are five phosphorylation sites within the C-terminal tail of FFA4 arranged in two clusters. These phosphorylation sites operate in concert with structural acidic residues to mediate the recruitment of arrestin-3 to FFA4.

Removing phosphorylation sites (TTSSS/AAAAA) or the structural elements (EDD/AAA) significantly reduced FFA4 interaction with arrestin-3 by 58 and 31%, respectively (Fig. 9). Combining the phosphorylation site mutations with the structural mutations (TTSSS/AAAA-EDD/AAA) reduced interaction with arrestin-3 almost entirely (11% retention) (Fig. 9). Together, these results indicate that phosphorylation and structural elements operate in combination in a largely additive fashion to regulate agonist-mediated arrestin-3 interactions with FFA4. Fig. 10 summarizes the impact of the phosphorylation and structural components on agonist-induced arrestin-3 recruitment to FFA4.

## DISCUSSION

The realization that free fatty acids can act as biologically active molecules through a specific family of GPCRs (19) has led to the need to understand the *in vivo* biology of this receptor family consisting of FFA1–4 as well as the modes of regulation and signal transduction. Here we have focused on the FFA4 receptor, which is responsive to long and medium chain free fatty acids (19) and reported to be involved in a range of biological responses, including the regulation of glucose homeostasis (11), lipogenesis (14), and inflammation (13). We report that, like many GPCRs, FFA4 is rapidly phosphorylated following agonist occupation and that this post-translational modification is involved in the recruitment of and interaction with arrestin-3 and mediates arrestin-dependent processes, such as receptor internalization. Importantly, we also identify structural elements within the C-terminal tail of the receptor that operate in concert with the phosphorylation sites to provide maximum recruitment of arrestin-3.

A recent report utilizing *in silico* phosphorylation site prediction algorithms and site-directed mutagenesis identified three phosphorylation sites (Thr^347^, Ser^350^, and Ser^357^) in the C terminus of FFA4 (20). Here, using a combination of mass spectrometry and mutagenesis, we confirm that these three sites are phosphorylated in response to agonist. We also identify two additional sites in the C-terminal tail, Thr^349^ and Ser^360^, the phosphorylation status of which is altered in response to agonist treatment. Using a phosphospecific antibody that we generated based on the mass spectrometry studies we show both that agonist-mediated phosphorylation at Thr^347^ and Ser^350^ is rapid, occurring within minutes of receptor activation, and is sustained and importantly that these same residues become phosphorylated whether the receptor is expressed in either CHO- or HEK293-based cell lines.

We further demonstrate that FFA4 can become phosphorylated by PKC in a manner that is sensitive to PKC inhibitors. Despite this, agonist-regulated (homologous) phosphorylation was not inhibited by PKC inhibitors, indicating that simple feedback involving FFA4 activation of PKC and subsequent receptor phosphorylation is not in operation at least in these cells. Rather, our data support a recent study that reports that agonist-mediated FFA4 phosphorylation occurs via a member(s) of the G protein-coupled receptor kinase family (20). It would seem, however, that although heterologous phosphorylation mediated by PKC activated by other G_q/11_-coupled receptors is possible the sites of this heterologous phosphorylation are not likely to be Thr^347^ and Ser^350^ as recently reported (20) because our phosphospecific antibodies directed against these sites did not detect any change in phosphorylation following PKC activation.

Sequential removal of the five phosphorylation sites in the C-terminal tail of FFA4 resulted in a progressive reduction in agonist-mediated recruitment of arrestin-3. These data might support the notion that it is the bulk negative charge on the C-terminal tail that is responsible for driving receptor/arrestin-3 interactions and not necessarily the precise sites of phosphorylation. That this is not the case is indicated by the redundant nature of phosphorylation at site Thr^349^, which appeared not to contribute to arrestin-3 interaction. This phosphorylation site is located within a cluster of three sites (Thr^347^, Thr^349^, and Ser^350^). It is possible that phosphorylation of only two of these three sites is sufficient to provide the contribution to arrestin-3 interaction provided by this cluster. This cluster of phosphorylation sites then operates in concert with serines Ser^357^ and Ser^360^ that make up the second cluster, together driving full arrestin-3 recruitment. Hence, it appears for the FFA4 receptor that it is not simply the bulk negative charge contributed by phosphorylation at the C-terminal tail that is important for arrestin-3 interaction but that the location of the sites of phosphorylation also plays a role. Such a scenario has been suggested for a number of other receptors, including the M_3_ muscarinic receptor (21), β_2_-adrenergic receptor (22), CXCR4 receptor (23), thyrotropin-releasing hormone receptor (24, 25), and the somatostatin 2A receptor (26), leading to the notion that there exists a phosphorylation bar code where the specific sequence of phosphorylation sites encodes for the signaling outcome of GPCRs (21, 22, 27). In the case of FFA4, it appears that phosphorylation of at least two of the three sites in cluster 1 (*i.e.* Thr^347^, Thr^349^, and Ser^350^) and both of the serines in cluster 2 (Ser^357^ and Ser^360^) are required to be phosphorylated to give maximal arrestin-3 recruitment.

These phosphorylation sites appear to operate in concert with structural elements consisting of the acidic residues Glu^341^, Asp^348^, and Asp^355^ to recruit arrestin-3. Interestingly, when all the phosphorylation sites were intact, the structural elements appeared to play less of a role in arrestin-3 recruitment. Hence, mutating residues Asp^348^ and Asp^355^ in a receptor where the phosphorylation sites were intact had limited impact on arrestin-3 recruitment. In this case, it is only when all three acidic residues were mutated that any impact on arrestin-3 recruitment was observed. However, in experiments using mutant FFA4 receptors where phosphorylation sites were removed, the structural elements appeared to play a more significant role. This may have important functional implications because recent x-ray crystal studies of the interaction of a phosphorylated C-terminal peptide derived from the V2 vasopressin receptor with an arrestin (arrestin-2 in this instance) have shown that there are multiple points of interaction between the receptor peptide and the arrestin (28). It has been proposed that the nature of these interactions determines the active conformation of arrestin (22, 28), which in turn determines the signaling outcome of the receptor-arrestin complex (22, 28). It is possible, therefore, that the structural elements in the C-tail of FFA4 (Glu^341^, Asp^348^, and Asp^355^) directly interact with arrestin and work in combination with phosphorylation sites that are regulated in response to agonist to recruit arrestin and possibly drive a particular arrestin conformation. Hence, the nature of the active conformation of arrestin and therefore the signaling outcome of the FFA4-arrestin complex might be determined by the ensemble of FFA4 phosphorylation sites in combination with the structural elements within the C-terminal tail.

It was also noticeable as mutations and truncations were introduced into the C-terminal tail of FFA4 that, as well as resulting in reduced efficacy of TUG-891 in the receptor/arrestin-3 interaction assay, this was accompanied by a decrease in agonist potency. This is despite the lack of effect on receptor/G protein interactions and is likely to reflect that interaction between a GPCR and an arrestin produces a high affinity, stable, agonist-binding form (29). This may also provide the basis for the slower association rate between such modified receptors and arrestin-3 observed in the kinetic analyses. Although only studied as a surrogate for the initiation of potential arrestin-3-mediated signaling, it was also notable that such modifications in FFA4 reduced but did not eliminate agonist-induced internalization of the receptor at least until the truncation was sufficiently extensive (*i.e.* FFA4-340) that agonist-induced interaction with the arrestin-3 construct was all but abrogated.

Understanding the combined role of FFA4 phosphorylation and structural elements in the recruitment and possible activation of arrestins provides a basis for the design of FFA4 ligands that might drive specific signaling outcomes. Such ligands have been termed biased agonists (30–32) and have the potential to drive specific physiological outcomes by preferentially engaging a defined subset of receptor signaling pathways. At the present time, there is very limited available synthetic ligand pharmacology at FFA4, and as such, the arrestin-3-uncoupled form of the receptor produced in these studies may prove useful in proof of concept studies for which signals in a physiological setting are downstream of engagement with arrestins rather than G protein-mediated pathways. The studies here show that FFA4 signaling via G_q/11_-mediated pathways is not dependent on the phosphorylation status of the receptor, whereas arrestin-3 recruitment is regulated by FFA4 phosphorylation. At this fundamental level, our data certainly point to the fact that an FFA4 ligand showing stimulus bias toward receptor phosphorylation/arrestin signaling would have potentially different physiological outcomes from that of a ligand showing G protein bias. This might be important, for example, if FFA4 was being targeted in inflammation, a response that appears in macrophages to be mediated by FFA4 signaling through arrestin-3 (13). It is possible, however, to extend the interpretation of our studies and predict that a more subtle form of stimulus bias might be possible. Ligands that drive different receptor phosphorylation patterns are beginning to emerge (21) and raise the possibility that FFA4 ligands could be identified that generate different phosphorylation ensembles (or bar codes). From the data presented in this study, one might predict that such ligands would differentially drive arrestin recruitment and possibly differential arrestin active conformations and in this way preferentially activate signaling and physiological outcomes. As the FFA4 receptor continues to be studied as a potential therapeutic target for a number of disease indications, including type II diabetes and inflammatory conditions (19), the design of ligands showing stimulus bias (33) might prove important in strategies to improve clinical efficacy and avoid adverse responses. The data provided in this study suggest that designing FFA4 ligands that can drive different patterns of receptor phosphorylation might provide a mechanism by which differential signaling through arrestin-3 might be achieved.

## Footnotes

4 The abbreviations used are:

GPCR: G protein-coupled receptor
BRET: bioluminescence resonance energy transfer
eYFP: enhanced yellow fluorescent protein
Rluc: *Renilla* luciferase
TEAB: triethylammonium bicarbonate
FFA: free fatty acid receptor
TUG-891: 3-(4-((4-fluoro-4′-methyl-[1,1′-biphenyl]-2-yl)methoxy)phenyl)propanoic acid.
